# 2-(4-Nitro­phen­yl)-1,3-dithiane

**DOI:** 10.1107/S1600536809001809

**Published:** 2009-01-17

**Authors:** Hoong-Kun Fun, Reza Kia, Annada C. Maity, Shyamaprosad Goswami

**Affiliations:** a X-ray Crystallography Unit, School of Physics, Universiti Sains Malaysia, 11800 USM, Penang, Malaysia; b Department of Chemistry, Bengal Engineering and Science University, Shibpur, Howrah 711 103, India

## Abstract

The nitro group in the title compound, C_10_H_11_NO_2_S_2_, is almost coplanar with the benzene ring, making a dihedral angle of 3.42 (8)°. The 1,3-dithiane ring adopts a chair conformation. The crystal structure is stabilized by inter­molecular C—H⋯O and C—H⋯π [C⋯*Cg* = 3.4972 (10) Å] inter­actions.

## Related literature

For hydrogen-bond motifs, see: Bernstein *et al.* (1995 ▶). For the calculation of ring puckering parameters, see: Cremer & Pople (1975 ▶). For related literature and applications see, for example: Goswami & Maity (2008 ▶); Fun *et al.* (2009 ▶).
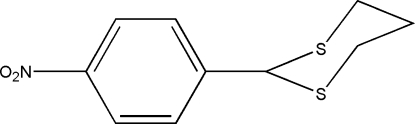


## Experimental

### 

#### Crystal data

C_10_H_11_NO_2_S_2_
*M* *_r_* = 241.32
Orthorhombic, 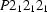

*a* = 8.7724 (1) Å
*b* = 10.2079 (1) Å
*c* = 11.9942 (1) Å
*V* = 1074.05 (2) Å^3^
*Z* = 4
Mo *K*α radiation
μ = 0.47 mm^−1^
*T* = 100 (1) K
0.46 × 0.22 × 0.08 mm

#### Data collection

Bruker SMART APEXII CCD area-detector diffractometer
Absorption correction: multi-scan (**SADABS**; Bruker, 2005 ▶) *T*_min_ = 0.813, *T*_max_ = 0.964
30643 measured reflections
4725 independent reflections
4511 reflections with *I* > 2σ(*I*)
*R*_int_ = 0.038

#### Refinement

*R*[*F* ^2^ > 2σ(*F* ^2^)] = 0.024
*wR*(*F* ^2^) = 0.061
*S* = 1.06
4725 reflections
136 parameters
H-atom parameters constrained
Δρ_max_ = 0.33 e Å^−3^
Δρ_min_ = −0.25 e Å^−3^
Absolute structure: Flack (1983 ▶), 2050 Friedel pairs
Flack parameter: 0.01 (4)

### 

Data collection: *APEX2* (Bruker, 2005 ▶); cell refinement: *SAINT* (Bruker, 2005 ▶); data reduction: *SAINT*; program(s) used to solve structure: *SHELXTL* (Sheldrick, 2008 ▶); program(s) used to refine structure: *SHELXTL*; molecular graphics: *SHELXTL*; software used to prepare material for publication: *SHELXTL* and *PLATON* (Spek, 2003 ▶).

## Supplementary Material

Crystal structure: contains datablocks global, I. DOI: 10.1107/S1600536809001809/tk2357sup1.cif

Structure factors: contains datablocks I. DOI: 10.1107/S1600536809001809/tk2357Isup2.hkl

Additional supplementary materials:  crystallographic information; 3D view; checkCIF report

## Figures and Tables

**Table 1:** Hydrogen-bond geometry (Å, °)

*D*—H⋯*A*	*D*—H	H⋯*A*	*D*⋯*A*	*D*—H⋯*A*
C6—H6*A*⋯O2^i^	0.93	2.59	3.3346 (12)	137
C1—H1*A*⋯*Cg*1^ii^	0.97	2.60	3.4972 (10)	154

Symmetry codes: (i) 
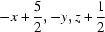
; (ii) 
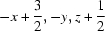
. *Cg*1 is the centroid of the benzene ring.

**Fig. 1.**
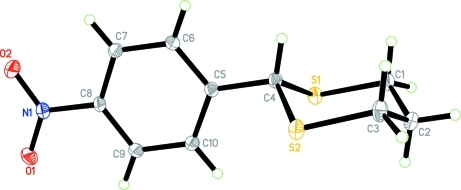
The molecular structure of (I), showing 50% probability displacement ellipsoids and the atomic numbering.

## supplementary crystallographic information

### Comment

Thioacetal protection of carbonyl groups is of paramount importance in synthetic organic chemistry and hence, the development of novel thionation reactions remains of great interest (Goswami & Maity, 2008; Fun *et al.,* 2009). In addition, thioacetals are also utilized as masked acyl anions or masked methylene functions in carbon-carbon bond forming reactions. Herein, we report the synthesis of 2-(4-nitro-phenyl)-[1,3]-dithiane (I) from 4-nitrobenzaldehyde using boron trifluoride etherate catalyst.

Compound (I), Fig. 1, comprises a single molecule in the asymmetric unit. The nitro group is almost coplanar with the benzene ring, making a dihedral angle of 3.42 (8)°. The thiacyclohexane ring adopts a chair conformation with the ring puckering parameters (Cremer & Pople, 1975) of Q = 0.7137 (8) Å, Θ = 173.96 (7)°, and Φ = 135.6 (7)°. The crystal structure is stabilized by intermolecular C—H···O and C—H···π interactions, Table 1.

### Experimental

To a stirred dichloromethane (50 mL) solution, maintained at 273 K, of 4-nitrobenzaldehyde (500 mg, 3.31 mmol) and boron trifluoride etherate (0.5 mL) was added dropwise 1,3-propanedithiol (450 mg, 4.1 mmol) over 15 min. The mixture was stirred at room temperature for 4 h and the progress of the reaction monitored by TLC. After completion of the reaction, NaHCO_3_ solution was added carefully at room temperature to neutralize the mixture which was then extracted with dichloromethane. The organic layer was dried (anhydrous Na_2_SO_4_) and the solvent removed under reduced pressure. The crude product was purified by column chromatography using silica gel with 10% ethyl acetate in petroleum ether as eluant to afford (I) (674 mg, 84%) as a colourless crystalline solid along with the other thiane derivatives.

### Refinement

All hydrogen atoms were positioned geometrically and refined with a riding model approximation with C—H = 0.93-0.98 Å, and with U_iso_ (H) = 1.2 U_eq_ (C).

### Crystal data

**Table:**

C_10_H_11_NO_2_S_2_	*F*(000) = 504
*M**_r_* = 241.32	*D*_x_ = 1.492 Mg m^−^^3^
Orthorhombic, *P*2_1_2_1_2_1_	Mo *K*α radiation, λ = 0.71073 Å
Hall symbol: P 2ac 2ab	Cell parameters from 9979 reflections
*a* = 8.7724 (1) Å	θ = 2.6–38.5°
*b* = 10.2079 (1) Å	µ = 0.47 mm^−^^1^
*c* = 11.9942 (1) Å	*T* = 100 K
*V* = 1074.05 (2) Å^3^	Block, colourless
*Z* = 4	0.46 × 0.22 × 0.08 mm

### Data collection

**Table:**

Bruker SMART APEXII CCD area-detector diffractometer	4725 independent reflections
Radiation source: fine-focus sealed tube	4511 reflections with *I* > 2σ(*I*)
graphite	*R*_int_ = 0.038
φ and ω scans	θ_max_ = 35.0°, θ_min_ = 2.6°
Absorption correction: multi-scan (*SADABS*; Bruker, 2005)	*h* = −14→14
*T*_min_ = 0.813, *T*_max_ = 0.964	*k* = −16→16
30643 measured reflections	*l* = −18→19

### Refinement

**Table:**

Refinement on *F*^2^	Secondary atom site location: difference Fourier map
Least-squares matrix: full	Hydrogen site location: inferred from neighbouring sites
*R*[*F*^2^ > 2σ(*F*^2^)] = 0.024	H-atom parameters constrained
*wR*(*F*^2^) = 0.061	*w* = 1/[σ^2^(*F*_o_^2^) + (0.0321*P*)^2^ + 0.1305*P*] where *P* = (*F*_o_^2^ + 2*F*_c_^2^)/3
*S* = 1.06	(Δ/σ)_max_ = 0.001
4725 reflections	Δρ_max_ = 0.33 e Å^−^^3^
136 parameters	Δρ_min_ = −0.25 e Å^−^^3^
0 restraints	Absolute structure: Flack (1983), 2050 Friedel pairs
Primary atom site location: structure-invariant direct methods	Flack parameter: 0.01 (4)

### Special details

**Table:**

Experimental. The low-temperature data was collected with the Oxford Cyrosystem Cobra low-temperature attachment.
Geometry. All esds (except the esd in the dihedral angle between two l.s. planes) are estimated using the full covariance matrix. The cell esds are taken into account individually in the estimation of esds in distances, angles and torsion angles; correlations between esds in cell parameters are only used when they are defined by crystal symmetry. An approximate (isotropic) treatment of cell esds is used for estimating esds involving l.s. planes.
Refinement. Refinement of F^2^ against ALL reflections. The weighted R-factor wR and goodness of fit S are based on F^2^, conventional R-factors R are based on F, with F set to zero for negative F^2^. The threshold expression of F^2^ > 2sigma(F^2^) is used only for calculating R-factors(gt) etc. and is not relevant to the choice of reflections for refinement. R-factors based on F^2^ are statistically about twice as large as those based on F, and R- factors based on ALL data will be even larger.

### Fractional atomic coordinates and isotropic or equivalent isotropic displacement parameters (Å^2^)

**Table:**

	*x*	*y*	*z*	*U*_iso_*/*U*_eq_	
S1	0.78978 (3)	0.06239 (2)	0.216265 (18)	0.01399 (5)	
S2	1.07695 (3)	0.22170 (2)	0.261209 (18)	0.01492 (5)	
O1	0.99728 (9)	0.26901 (8)	−0.32568 (6)	0.01819 (14)	
O2	1.12536 (10)	0.08795 (8)	−0.33808 (6)	0.02137 (15)	
N1	1.05536 (9)	0.17161 (8)	−0.28383 (7)	0.01391 (13)	
C1	0.80248 (11)	0.03483 (9)	0.36543 (8)	0.01526 (16)	
H1A	0.7009	0.0182	0.3941	0.018*	
H1B	0.8631	−0.0431	0.3785	0.018*	
C2	0.87227 (11)	0.14837 (10)	0.43022 (8)	0.01481 (15)	
H2A	0.8157	0.2276	0.4135	0.018*	
H2B	0.8618	0.1312	0.5094	0.018*	
C3	1.03975 (11)	0.17104 (11)	0.40367 (8)	0.01636 (17)	
H3A	1.0954	0.0907	0.4182	0.020*	
H3B	1.0790	0.2376	0.4538	0.020*	
C4	0.99151 (10)	0.08515 (9)	0.18616 (7)	0.01235 (15)	
H4A	1.0463	0.0046	0.2055	0.015*	
C5	1.01013 (10)	0.11049 (9)	0.06296 (7)	0.01189 (14)	
C6	1.09657 (10)	0.02450 (9)	−0.00171 (7)	0.01299 (15)	
H6A	1.1440	−0.0468	0.0318	0.016*	
C7	1.11247 (10)	0.04450 (9)	−0.11583 (8)	0.01293 (15)	
H7A	1.1690	−0.0131	−0.1593	0.016*	
C8	1.04185 (10)	0.15252 (9)	−0.16301 (7)	0.01169 (14)	
C9	0.95653 (11)	0.24152 (9)	−0.10131 (8)	0.01406 (16)	
H9A	0.9114	0.3138	−0.1351	0.017*	
C10	0.94071 (11)	0.21912 (9)	0.01268 (7)	0.01455 (15)	
H10A	0.8836	0.2768	0.0558	0.017*	

### Atomic displacement parameters (Å^2^)

**Table:**

	*U*^11^	*U*^22^	*U*^33^	*U*^12^	*U*^13^	*U*^23^
S1	0.01279 (8)	0.01716 (10)	0.01201 (9)	−0.00234 (7)	−0.00105 (7)	−0.00003 (8)
S2	0.01512 (9)	0.01748 (10)	0.01216 (9)	−0.00450 (7)	0.00056 (7)	−0.00083 (7)
O1	0.0228 (3)	0.0179 (3)	0.0138 (3)	0.0016 (3)	−0.0017 (3)	0.0050 (3)
O2	0.0286 (4)	0.0219 (4)	0.0136 (3)	0.0056 (3)	0.0052 (3)	−0.0013 (3)
N1	0.0149 (3)	0.0152 (3)	0.0117 (3)	−0.0013 (3)	0.0008 (3)	0.0010 (3)
C1	0.0167 (4)	0.0169 (4)	0.0121 (4)	−0.0023 (3)	0.0021 (3)	0.0002 (3)
C2	0.0148 (4)	0.0183 (4)	0.0113 (4)	−0.0002 (3)	0.0016 (3)	−0.0019 (3)
C3	0.0147 (4)	0.0236 (4)	0.0108 (4)	−0.0019 (3)	−0.0012 (3)	−0.0010 (3)
C4	0.0133 (3)	0.0137 (4)	0.0100 (3)	0.0006 (3)	−0.0004 (3)	0.0001 (3)
C5	0.0127 (3)	0.0127 (3)	0.0103 (3)	0.0002 (3)	−0.0003 (3)	0.0006 (3)
C6	0.0142 (4)	0.0128 (3)	0.0120 (3)	0.0023 (3)	0.0001 (3)	0.0017 (3)
C7	0.0130 (3)	0.0134 (4)	0.0124 (3)	0.0009 (3)	0.0007 (3)	−0.0006 (3)
C8	0.0130 (3)	0.0131 (4)	0.0090 (3)	−0.0012 (3)	0.0005 (3)	0.0008 (3)
C9	0.0169 (4)	0.0130 (4)	0.0123 (4)	0.0025 (3)	0.0000 (3)	0.0015 (3)
C10	0.0182 (4)	0.0139 (4)	0.0116 (3)	0.0034 (3)	0.0007 (3)	0.0000 (3)

### Geometric parameters (Å, °)

**Table:**

S1—C1	1.8145 (9)	C3—H3B	0.9700
S1—C4	1.8210 (9)	C4—C5	1.5090 (12)
S2—C3	1.8148 (10)	C4—H4A	0.9800
S2—C4	1.8208 (9)	C5—C6	1.3953 (13)
O1—N1	1.2248 (10)	C5—C10	1.4016 (13)
O2—N1	1.2368 (11)	C6—C7	1.3910 (13)
N1—C8	1.4670 (11)	C6—H6A	0.9300
C1—C2	1.5238 (13)	C7—C8	1.3855 (12)
C1—H1A	0.9700	C7—H7A	0.9300
C1—H1B	0.9700	C8—C9	1.3905 (13)
C2—C3	1.5210 (13)	C9—C10	1.3930 (13)
C2—H2A	0.9700	C9—H9A	0.9300
C2—H2B	0.9700	C10—H10A	0.9300
C3—H3A	0.9700		
			
C1—S1—C4	98.95 (4)	C5—C4—S1	108.74 (6)
C3—S2—C4	99.98 (4)	S2—C4—S1	113.56 (5)
O1—N1—O2	123.46 (8)	C5—C4—H4A	108.8
O1—N1—C8	118.63 (8)	S2—C4—H4A	108.8
O2—N1—C8	117.91 (8)	S1—C4—H4A	108.8
C2—C1—S1	114.18 (6)	C6—C5—C10	119.65 (8)
C2—C1—H1A	108.7	C6—C5—C4	119.69 (8)
S1—C1—H1A	108.7	C10—C5—C4	120.66 (8)
C2—C1—H1B	108.7	C7—C6—C5	120.61 (8)
S1—C1—H1B	108.7	C7—C6—H6A	119.7
H1A—C1—H1B	107.6	C5—C6—H6A	119.7
C3—C2—C1	113.39 (8)	C8—C7—C6	118.28 (8)
C3—C2—H2A	108.9	C8—C7—H7A	120.9
C1—C2—H2A	108.9	C6—C7—H7A	120.9
C3—C2—H2B	108.9	C7—C8—C9	122.91 (8)
C1—C2—H2B	108.9	C7—C8—N1	118.23 (8)
H2A—C2—H2B	107.7	C9—C8—N1	118.85 (8)
C2—C3—S2	114.47 (6)	C8—C9—C10	117.95 (8)
C2—C3—H3A	108.6	C8—C9—H9A	121.0
S2—C3—H3A	108.6	C10—C9—H9A	121.0
C2—C3—H3B	108.6	C9—C10—C5	120.59 (8)
S2—C3—H3B	108.6	C9—C10—H10A	119.7
H3A—C3—H3B	107.6	C5—C10—H10A	119.7
C5—C4—S2	107.96 (6)		
			
C4—S1—C1—C2	60.04 (8)	C4—C5—C6—C7	178.61 (8)
S1—C1—C2—C3	−66.54 (10)	C5—C6—C7—C8	0.73 (13)
C1—C2—C3—S2	64.81 (10)	C6—C7—C8—C9	0.18 (14)
C4—S2—C3—C2	−57.45 (8)	C6—C7—C8—N1	−178.43 (8)
C3—S2—C4—C5	179.49 (6)	O1—N1—C8—C7	−177.77 (8)
C3—S2—C4—S1	58.85 (6)	O2—N1—C8—C7	2.17 (12)
C1—S1—C4—C5	−179.95 (6)	O1—N1—C8—C9	3.56 (12)
C1—S1—C4—S2	−59.74 (6)	O2—N1—C8—C9	−176.50 (9)
S2—C4—C5—C6	117.37 (8)	C7—C8—C9—C10	−0.79 (14)
S1—C4—C5—C6	−119.01 (8)	N1—C8—C9—C10	177.81 (8)
S2—C4—C5—C10	−63.01 (10)	C8—C9—C10—C5	0.50 (14)
S1—C4—C5—C10	60.60 (10)	C6—C5—C10—C9	0.38 (14)
C10—C5—C6—C7	−1.01 (14)	C4—C5—C10—C9	−179.24 (9)

### Hydrogen-bond geometry (Å, °)

**Table:**

*D*—H···*A*	*D*—H	H···*A*	*D*···*A*	*D*—H···*A*
C6—H6A···O2^i^	0.93	2.59	3.3346 (12)	137
C1—H1A···Cg1^ii^	0.97	2.60	3.4972 (10)	154

Symmetry codes: (i) −*x*+5/2, −*y*, *z*+1/2; (ii) −*x*+3/2, −*y*, *z*+1/2.

## References

[bb1] Bernstein, J., Davis, R. E., Shimoni, L. & Chang, N.-L. (1995). *Angew. Chem. Int. Ed. Engl* **34**, 1555-1573.

[bb2] Bruker (2005). *APEX2*, *SAINT* and *SADABS* Bruker AXS Inc., Madison, Wisconsin, USA.

[bb3] Cremer, D. & Pople, J. A. (1975). *J. Am. Chem. Soc.* **97**, 1354–1358.

[bb4] Flack, H. D. (1983). *Acta Cryst.* A**39**, 876–881.

[bb5] Fun, H.-K., Kia, R., Maity, A. C. & Goswami, S. (2009). *Acta Cryst.* E**65**, o173. 10.1107/S1600536808042864 PMC2968084 21581630

[bb6] Goswami, S. P. & Maity, A. C. (2008). *Tetrahedron Lett.* **49**, 3092–3096.

[bb7] Sheldrick, G. M. (2008). *Acta Cryst.* A**64**, 112–122. 10.1107/S0108767307043930 18156677

[bb8] Spek, A. L. (2003). *J. Appl. Cryst.* **36**, 7–13.

